# The pre-ejection period is a highly stress dependent parameter of paramount importance for pulse-wave-velocity based applications

**DOI:** 10.3389/fcvm.2023.1138356

**Published:** 2023-02-15

**Authors:** Niklas Pilz, Andreas Patzak, Tomas L. Bothe

**Affiliations:** Institute of Translational Physiology, Charité–Universitätsmedizin Berlin, Corporate Member of Freie Universität Berlin and Humboldt-Universität zu Berlin, Berlin, Germany

**Keywords:** pre-ejection period, pulse wave velocity, blood pressure, cuff-less blood pressure measurement, impedance-cardiography

## Abstract

**Purpose:**

The pulse-wave-velocity, is used for indirect, cuff-less, continuous blood pressure estimation. It is commonly detected by measuring the time delay between a defined point in an ECG and the arrival of the peripheral pulse wave (e.g., oxygen saturation sensor). The period between electrical stimulation of the heart (ECG) and actual blood ejection from the heart is called the pre-ejection period (PEP). This study aims at characterizing the PEP under mental and physical stress with focus on its relations to other cardiovascular parameters such as heart rate and importance for blood pressure (BP) estimation.

**Methods:**

We measured the PEP in 71 young adults at rest, under mental (TSST) and physical stress (ergometer) *via* impedance-cardiography.

**Results:**

The PEP is highly dependent on mental and physical load. It is strongly correlated with indicators of sympathetic strain (*p* < 0.001). At rest (mean 104.5 ms), the PEP shows a high interindividual variability but small intraindividual variability. Mental stress decreases the PEP by 16% (mean 90.0 ms) while physical stress halves PEP (mean 53.9 ms). The PEP does correlate differently with heart rate under differing circumstances (rest: *R*^2^ 0.06, mental stress: *R*^2^ 0.29, physical stress: *R*^2^ 0.65). Subsequently, using PEP and heart rate enables the discrimination of rest, mental and physical strain with a positive predictive value of 93%.

**Conclusion:**

The PEP is a cardiovascular parameter with large interindividual variability at rest and subject-depended dynamic under load which is of great importance for ECG-based pulse-wave-velocity (PWV) determination. Considering its variability and large impact on the pulse arrival time, PEP is a crucial factor in PWV based BP estimation.

## Introduction

The pulse-wave-velocity (PWV) is a widely used parameter for describing, and diagnosing, the state of a patient’s cardiovascular system (1–4). Further, the correlation of peripheral PWV to blood pressure (BP) (5) led to its application for continuous, cuff-less, blood pressure (BP) measurement (6).

There are multiple options for determining the PWV (7). However, applications of continuous and cuff-less BP measurement predominantly rely on an electro-cardiogram (ECG) and peripheral detection of the pulse pressure to derive the peripheral PWV (8–10).

**FIGURE 1:**
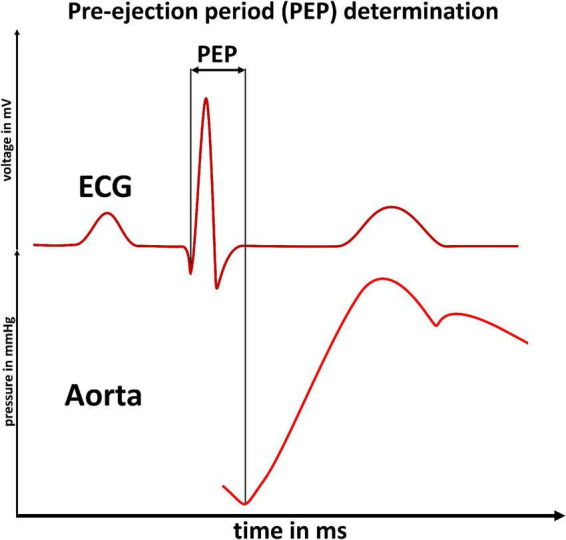
Pre-ejection period determination: Illustration of the pre-ejection period (PEP) as the time delay between an ECG’s Q-wave (upper panel) and the start of blood ejection (increased flow in the aorta, lower panel). The PEP cannot be detected by ECG based measurements of the pulse-wave-velocity.

The PWV is the speed of propagation of the blood ejections inertia over the vessel walls, generated by the heart (11). Subsequently, the PWV describes a mechanical phenomenon, with well characterized dependencies to vessel stiffness. Unfortunately, for all its benefits of technical, real-world applicability, determining the PWV *via* an ECG signal poses a major limitation: The ECG measures electrical processes in the heart and cannot detect the true, mechanical start of blood ejection from the heart (the true start time for PWV calculation). This time delay between an ECG’s Q-wave and the actual blood ejection from the heart is called the pre-ejection period (PEP) (12) (Figure 1).

There are various options for measuring the PEP: Intraarterial measurement (13), ECG-coupled sonography (14) or *via* impedance cardiography (15, 16). A recent work showed little intra-individual PEP variability at rest (17). From this it could be concluded that PEP can be neglected for PWV based BP measurement. On the contrary, works from the field of psychobiology have shown that PEP does change under stress (18–20). Moreover, the degree of PEP variation under stress seems to be highly variable between different individuals (21, 22).

Subsequently, PEP’s importance for PWV based BP measurement remains unclear. In this study, we aim at answering the question whether PEP needs to be considered for precise continuous BP measurement. Further, we investigate if PEP can be estimated and therefore addressed indirectly–or should be measured.

## Materials and methods

### Ethical approval

The study was approved by the local ethics committee (ethics committee Charité–Universitätsmedizin Berlin, approval-number: EA4/051/21). We registered the study at Charité–Universitätsmedizin Berlin’s clinical trial register before the start of data collection (ePA: 3000224).

### Setup and devices

We used the CardioScreen^®^ 1000 (medis Medizinische Messtechnik GmbH, Ilmenau, Germany) device to perform impedance cardiography. The device has been validated (as bedside monitor, named *niccomo™-*monitor) (16, 23–25). We recorded all parameters provided by the device, including the PEP, heart rate, left ventricular ejection time, and Heather index. The Heather index is calculated as a combination of acceleration and velocity of blood flow (both as index values, relative to body surface area) and is a parameter of impedance cardiography said to represent contractility and overall sympathetic tone (26). For BP measurement, we used the validated cuff-based OnTrak 90227 device (Spacelabs^®^ Healthcare) (27). For ensuring valid cuff-based measurement, we recorded the cuff’s pressure curves *via* a Y-connection, and recorded data *via* a SOMNOtouch™ NIBP (SOMNOmedics GmbH).

The devices were time synchronized in a two-step process. The CardioScreen^®^ and SOMNOtouch™ devices were both initiated to within second precision during device setup. As both devices recorded an ECG, we were then able to synchronize the signals with millisecond precision.

We used a Ergometrics 900 L (ergoline GmbH, Bitz, Germany) recumbent bike ergometer with 60° inclination to enable controllable physical load while minimizing upper body movement. This is of utmost importance for the 8-lead cardio impedance device and during cuff-based BP measurement. The experimental setup, combined with quality assessment of cuff pressure curves, allowed us to maximize the quality of measurements included in the final analysis. We time synchronized all devices.

### Procedure

**FIGURE 2:**
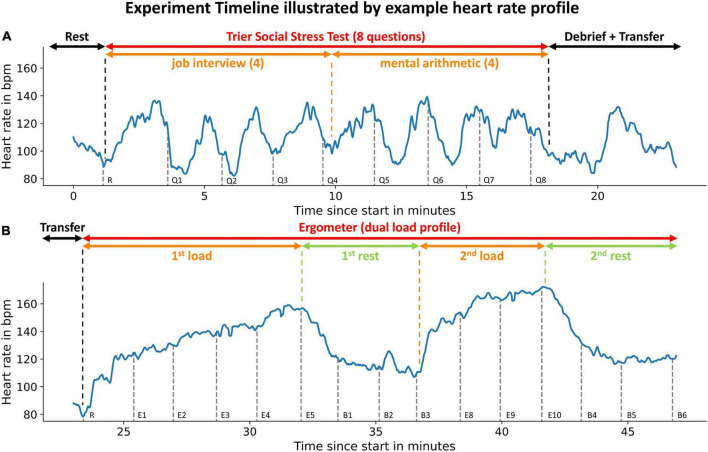
Experiment timeline illustrated by example heart rate profile: Phases of the experiments are indicated by the uppermost arrows. Subsets of experimental phases are indicated by the second row of arrows. An example heart rate profile is displayed in blue. Gray dashed vertical lines indicate the initiation of blood pressure measurements. The measurement instance is denoted to the right of the dashed line. The participant did not reach load-phases E6 + E7 (see Figure 4). The upper panel **(A)** illustrates the first experimental phase (mental load), the lower panel **(B)** the second phase (physical load).

We performed impedance-cardiography and cuff-based BP measurement at rest and under mental and physical load in 71 young and healthy adults. After welcoming subjects and receiving their written consent, we proceeded with the experiment. We asked the participants to sit on a chair behind a desk with all devices attached. After 2 min at total rest, we initiated the impedance cardiography and performed the first BP measurement. This measurement was taken to record at rest (baseline) measurements of BP and impedance cardiography results which we needed to show intraindividual changes in the following analysis. Subsequently, the subjects each went through two experimental phases (Figure 2).

### Trier Social Stress Test

We used an adapted version of the Trier Social Stress Test (TSST) to submit the participants to mental stress (28, 29) (Figure 2A). The adapted TSST consisted of four questions from a simulated job application interview and four subsequent mental arithmetic questions. After every question a BP measurement was taken. A detailed description can be found in the Supplementary material.

After the TSST was completed, we debriefed the subjects. We focused on an extensive and effective debrief for two reasons: Most importantly, we wanted to ensure an overall positive experience for all participants. Further, an effective debrief ensured a close to the mental load induced by the TSST and allowed us to perform an examination of a solely physical stress reaction of PEP thereafter.

### Bike ergometer

After the debrief, we transferred the participants to a bike ergometer (Figure 2B). We were able to only interrupt measurement for a few minutes as we performed both experimental phases in the same room.

Following the transfer, we waited until participants reached their resting heart rate before starting the bimodal exercise profile. Participants were instructed to tread steadily on the bike ergometer (60 rpm). We increased the physical load in weight adapted steps (0.4 x body mass in kg). After 1 min, we initiated a BP measurement while the participants continued treading steadily. To ensure optimal conditions for BP measurement, we rested the participants’ arm on an arm support and instructed the participants to relax the arm. Subsequently, we increased the physical load by one step after the BP measurement was finished and repeated the procedure. We increased the load until participants exceeded 80% of their calculated maximum heart rate (220–age in years). Followingly, the participants were instructed to stop treading and we performed three resting BP measurements (starting 60 s after finishing the measurement before). Then, we instructed the now rested participants to begin treading again, starting on the second to last step they had reached during the first physical load. Similarly, to the first physical load phase, we initiated a BP measurement after 60 s and raised the load by one step after the BP measurement had concluded. We then performed two BP measurements (each initiated 60 s after the last BP measurement had ended) on this load stage. Therefore, we did not increase load beyond the maximum load reached in the first phase. Afterwards, we performed three BP measurements during a second rest phase which concluded the experiment.

### Data processing and statistical analysis

We performed a quality check for all impedance cardiography datasets, excluding measurement errors defined as abnormal changes in heart rate (change > 30% in less than 3 s). In line with manufacturer recommendations, we averaged the beat-to-beat data over four heartbeats.

Blood pressure measurements were assessed for undisturbed pressure curves. We excluded measurements when there was an increase of cuff pressure of more than 8 mmHg during cuff deflation.

To decide whether to use the ECG’s Q-wave (physiological beginning of PEP = start of ventricular depolarization) or the R-wave (easier detection), we compared the PEP derived from both starting points using a correlation analysis and analyzed the changes in the Q-R-time and its dependency to mental or physical load.

We analyzed changes of PEP during the TSST and ergometer load separately by modeling a mixed linear model (IBM SPSS Statistics 27). For Regression analyses, we performed linear regression and non-linear, *R*^2^-calculation *via* Scikit-learn (30). To find differences in PEP’s behavior under different circumstances, we calculated a mixed linear model and adjusted for the heart rate as a covariate factor. Subsequently, we trained a k-nearest-neighbor classifier with patient mean values for rest, TSST and bike ergometer load. In accordance with best practices of Machine Learning we performed a strict separation of training and test data. We tested the classifier using a subject-dependent train-test split. This means that all three data-points of any given patient had to be either in the train or the test group, therefore reducing the change for data-leakage. We retrieved our results by evaluating the classifier in an 80/20 k-fold (equates to fivefold) evaluation scheme. We averaged the results (positive predictive value/sensitivity) over all three outcomes (rest, mental load, physical load) and over all k-fold iterations to provide an aggregate estimation of the classifiers performance.

To illustrate patient specific differences at rest and under mental and physical load, we visualized data at rest measurement, first TSST question and last ergometer step (maximum load) in a boxplot. We analyzed the differences *via* a one-way ANOVA. The statistical analysis was performed in close collaboration with Charité’s Institute of Biometry and Clinical Epidemiology. We used the conservative Bonferroni correction for all multi-group comparisons.

To estimate the effect of neglecting or estimating the PEP, we analyzed a previously published and often-cited relation between PWV and BP (6). We applied the relation for a standard human (1.80 m) and translated the relation to a pulse-arrival-time (PAT, time from ECG Q-wave to arrival of the pulse wave in the periphery) vs. BP relation. Thereafter, we used our data to estimate the PEP’s proportion of the PAT for different BP levels (regression) and subtracted the PEP to receive the pulse-transit-time (PTT = PAT–PEP). We then analyzed the PEP estimation uncertainty (intra- and interindividual PEP variability at similar BP levels) and calculated confidence intervals for one SD (67% of values) and two SDs (95% of values) for PTT. Applying this relation, we were able to determine the BP measurement estimation uncertainty caused by either neglecting or estimating the PEP for PWV based BP measurement.

## Results

### Dataset composition

We conducted the experiment with 71 young and healthy adults (Table 1).

**TABLE 1:** Dataset composition.

	Total (*N* = 71)		Male (*N* = 34)		Female (*N* = 37)	
	Mean	SD	Mean	SD	Mean	SD
Age in years	21.9	2.8	21.4	2.3	22.3	3.2
Height in cm	175.2	9.9	183.3	7.1	167.6	4.9
Weight in kg	68.8	13.0	79.2	10.1	59.2	6.2

Two participants only completed part two of the experiment (physical load) while one participant had to terminate the experiment due to feeling unwell after the TSST. In one case, a technical problem with the impedance cardiography prohibited analysis of the retrieved data for all parts of the experiment. This led to valid results for 68 participants for phase one (TSST) and 69 for phase two (ergometer).

### Stating point of PEP

The analysis showed a strong correlation (*p* < 0.001) between PEP starting from the Q-wave and PEP starting from the R-wave of *r* = 0.97. Further, the Q-R-time (time interval between ECG’s Q- and R-wave) showed no correlation to the increase of heart rate under load (*p* > 0.05) and a mean standard deviation of <4 ms.

### PEP variability under resting conditions

**FIGURE 3:**
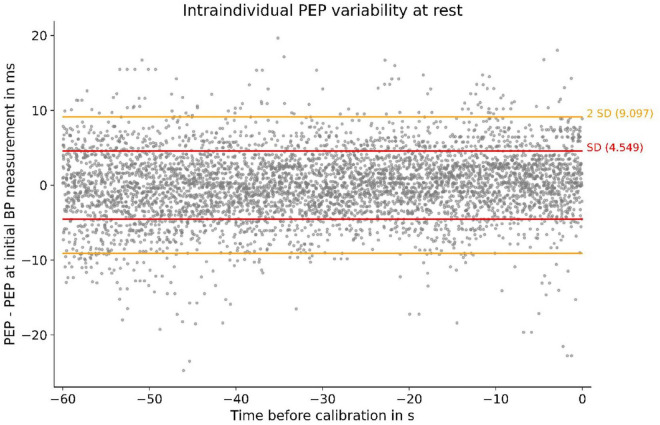
Individual PEP variability at rest of all subjects. Measurements were obtained in the 60 s before calibration: SD, standard deviation; PEP–PEP at first BP measurement (at rest) depicted for 60 s before the BP measurement. The SD of 4.5 ms symbolizes small intraindividual PEP variability at rest.

Under resting conditions, we observed very little intraindividual PEP variability. Compared to their PEP at the first BP measurement (at rest), participants’ PEP values in the 60 s before the BP measurement (at total rest) deviated only marginally (Figure 3). The resting PEP showed a sex difference of −6.2 ms (*p* = 0.043) for females and weak but present correlations with weight (R^2^ = 0.13, *p* = 0.004) and height (R^2^ = 0.08, *p* = 0.033).

### PEP under mental and physical load

**FIGURE 4:**
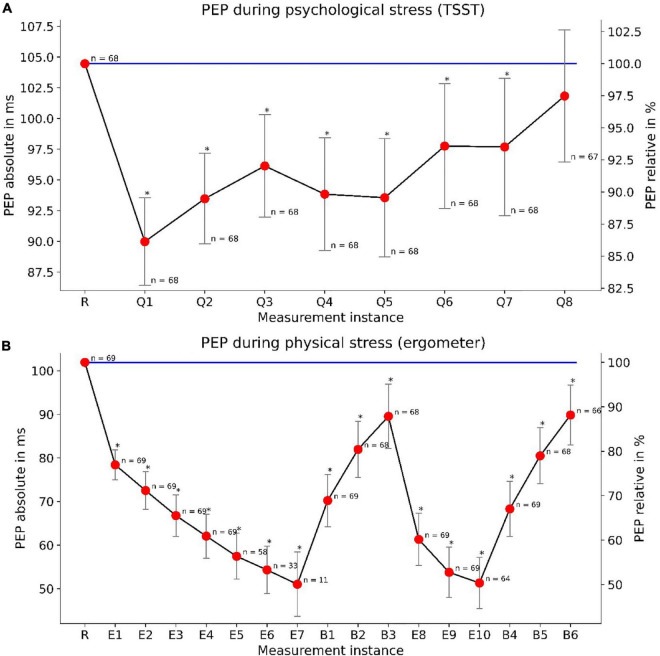
PEP changes under mental (TSST) and physical (ergometer) load: The upper panel **(A)** depicts the PEP reactivity during the TSST. “R” marks the measurement at rest while “Q1”–“Q8” demark TSST questions. The lower panel **(B)** depicts PEP reactivity and load-response dependency under physical stress. “R” marks a measurement at rest, “E1”–“E10” demark measurements under physical load and “B1”–“B6” demark measurements during the break-period. We depicted PEP measurements taken at the end of their according BP measurements. Declining *n*-numbers are due to dropouts (during TSST) or reaching the maximum heart rate at earlier load steps (ergometer). Whiskers show confidence intervals (*p* < 0.05) for relative PEP values. The absolute variance of PEP is more clearly depicted in Figure 8.

The PEP changes significantly under psychological and physical stress. The TSST induced a noticeable reduction of PEP. In detail, we discovered a habituation effect to PEP’s response to psychological stress. The first TSST question reduces the PEP by about 14%. This effect got smaller during the TSST, leading to a non-significant difference for the last question (Figure 4A).

For physical load, we found an even more profound reduction of PEP. With increasing load, the PEP continues to fall up to a reduction of 50%. PEP showed a load-response behavior, with steadily decreasing PEP under increasing load and vice versa, steadily increasing PEP during rest. We observed these effects during both load/rest cycles of the bimodal load profile (Figure 4B). The results indicate a clear load-response relationship between physical load and PEP reduction compared to values at rest. Moreover, mental, and physical stress showed to not influence PEP symmetrically. While PEP did decrease under both mental and physical load, PEP modulation by physical exercise was much more pronounced.

### Correlation to cardiovascular parameters

**FIGURE 5:**
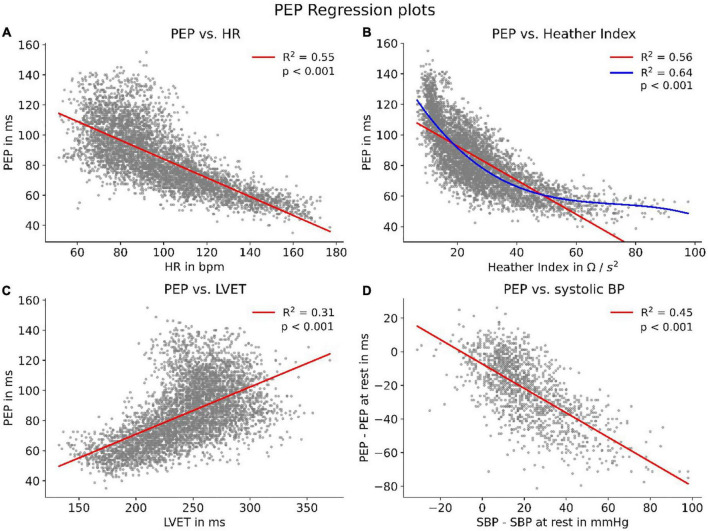
HR, heart rate; LVET, left-ventricular-ejection-time; Correlations of PEP with parameters connected to sympathetic tone. To enhance visibility, panels **(A–C)** show scatters of 5,000 randomly sampled datapoints while correlation coefficients were calculated for the whole dataset. Panel **(B)** features an additional polynomial fit (blue) to account for the non-linear data distribution. Panel **(D)** shows the correlation between PEP and SBP compared to their respective values at rest.

We were able to show strong correlations between PEP and other cardiovascular parameters, all of which said to be connected to sympathetic tone. The strongest correlation (*R*^2^ = 0.64, *p* < 0.001) presented itself for PEP and Heather index. Further, we found strong correlations (*p* < 0.001) with heart rate (*R*^2^ = 0.55), left-ventricular-ejection-time (*R*^2^ = 0.31) and systolic BP (SBP) (*R*^2^ = 0.45) (Figure 5).

### Load dependent correlation to heart rate

**FIGURE 6:**
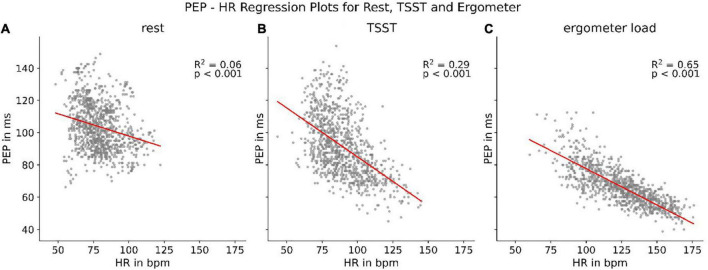
Regression between heart rate (HR) and PEP at rest **(A)**, during TSST **(B)**, and treading **(C)** the bike ergometer.

Although PEP shows a strong correlation with parameters related to the activity of the autonomous nervous system, we discovered that this correlation is highly dependent on the current load a participant was under. Splitting the PEP/heart rate correlations up into correlations at rest, during the TSST and during treading the bike ergometer showed clear discrepancies. While all resulted in statistically present correlations (*p* < 0.001), the correlation strength differed heavily (Figure 6). The results indicate an increase of synchronization between heart rate and PEP with increasing sympathetic activation.

### Determining load state *via* PEP and heart rate (k-NN)

**FIGURE 7:**
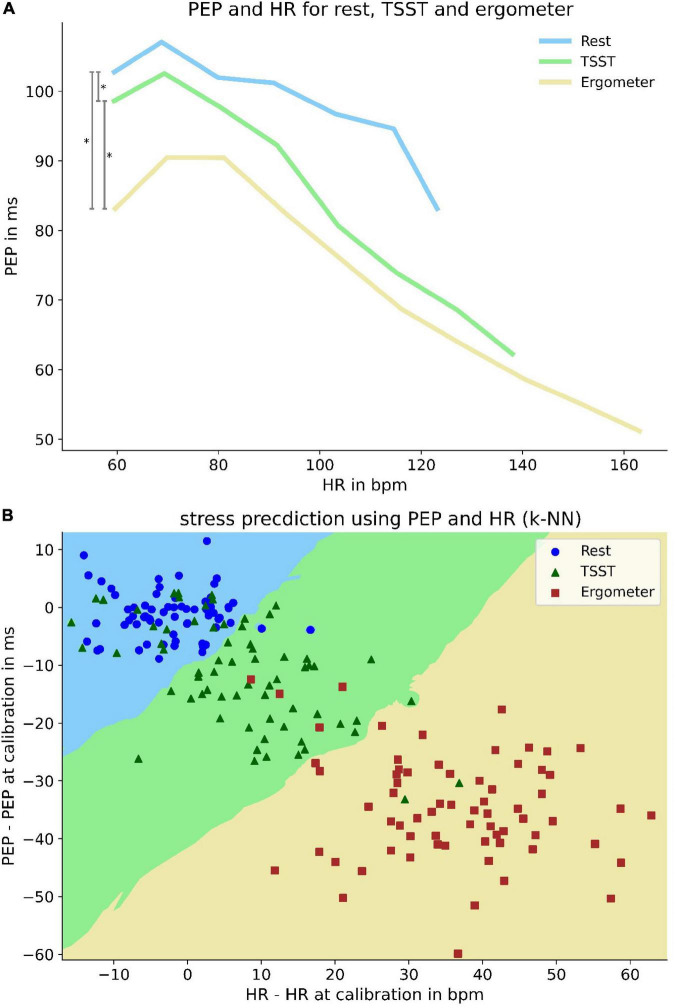
Differences of PEP at same heart rate and its predictive power for current load. Upper panel **(A)**: Differences of PEP at same heart rates at rest and under mental and physical load. To enhance visibility, PEP values are averaged over a heart rate range of 12 bpm. Lower pane **(B)** l: k-nearest-neighbor predictor (*k* = 13) of load-state based on heart rate and PEP deviation from measurement at rest. Decision boundaries are marked by colored areas in the plot. Bisecting decision boundaries represent a coequal importance of PEP and HR for predictive power (e.g., solely heart rate based predictor would show vertical decision boundaries). Scattered elements represent patient specific mean values under rest (blue circle), mental (green triangle) and physical load (red square).

Not only did we find differences in PEP correlation with heart rate under differing circumstances but were also able to identify differing PEP behavior for the same heart rate during rest, TSST and physical load. From rest to TSST to physical load, the PEP decreased during similar heart rates (*p* < 0.001) (Figure 7A). This indicated that there is predictive power for the current load (rest, mental or physical load) when combining information from heart rate and PEP. Followingly, we trained a k-nearest-neighbor classifier with subject specific mean PEP and heart rate values (as delta to their single measurement value at total rest). The classifier retrieved a positive predictive value of 93% and a sensitivity of 92% for identifying load states based on current PEP and heart rate values (Figure 7B).

### Interindividual variance and dynamic of PEP

**FIGURE 8:**
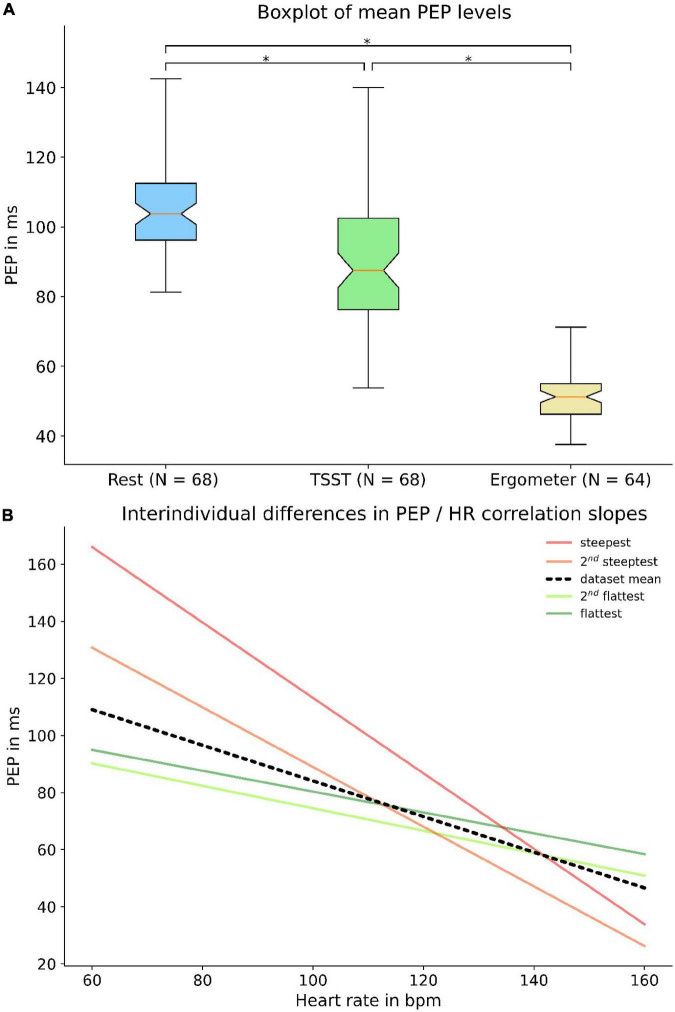
Interindividual variance of PEP: The upper panel shows a boxplot **(A)** of individual subject PEP values at rest, at the first question of TSST and the last step of bike ergometer treading. Broad quartiles and long whiskers symbolize a large interindividual variability. The lower panel shows interindividual differences in regression slopes **(B)** between heart rate and PEP. The black line indicates the dataset mean while the green and red lines represent the flattest and steepest slopes, respectively. * = *p* < 0.05.

In addition to the modulated dependency of PEP to heart rate, we were able to reveal large interindividual discrepancies in PEP at rest and of PEP behavior under load. An analysis of values derived at total rest, the first question of TSST (maximum mental load) and the last step of bike ergometer treading (maximum physical load) showed interindividual differences of almost 50% during rest and physical load and even more profound differences during the TSST (Figure 8A). The large discrepancy during the TSST was likely partly due to differences in response to mental stress. Not only resting values showed a large interindividual variability, but there were also large differences in PEP’s dynamic as well. Our analysis of individual regression slopes (how much does PEP change for every change of heart rate) showed large differences between subjects (Figure 8B).

### PEP’s importance for PWV-based BP estimation

**FIGURE 9:**
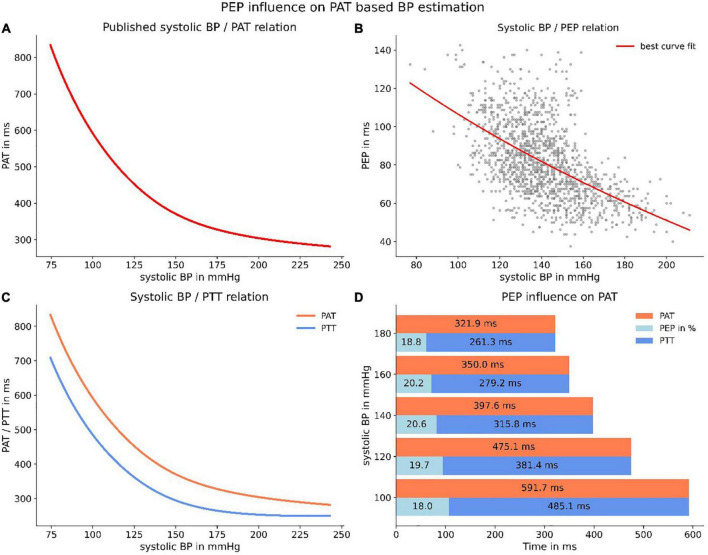
Applying PEP to a published PWV-BP relation. Panel **(A)** depicts the relation, transformed to a PAT/BP relation for a “standard” human (1.80 m). Panel **(B)** represents the best polynomial fit for our data of PEP and SBP. Panel **(C)** depicts the resulting PTT-BP relation (PTT = PAT–PEP) when correcting PAT for PEP. The resulting line (blue) is the purest representation of a true PWV-BP relationship. Panel **(D)** highlights the (on average) constant proportion of PEP in PAT.

Following these analyses, we were able to apply the findings to a formerly published application of measuring the BP *via* PWV measurement (31). We transformed the relation published in the paper to a PAT-BP relation for a “standard” human (1.80 m) in accordance with the authors recommendations. The resulting relationship matched the real-world scenario in which the PAT (ECG Q-wave to arrival of the pulse wave in the periphery) is measured and SBP is derived (Figure 9A). We then used our data to calculate a polynomial relation between SBP and PEP to serve as best guess for estimating the PEP (Figure 9B). Subsequently, we inferred a relationship between SBP and PTT (PAT–PEP) which is the true representation of a PWV-BP model, because the effect of PEP is eliminated (Figure 9C). Further, we were able to show that on average the proportion of PEP in PAT is putatively constant (Figure 9D).

**FIGURE 10:**
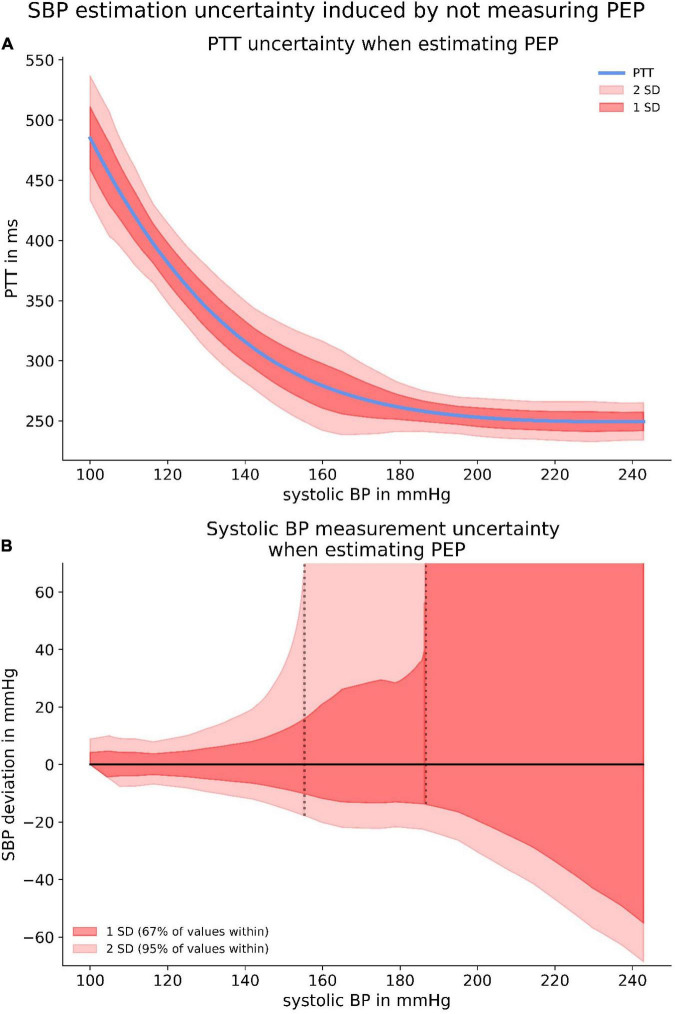
The upper panel **(A)** depicts confidence intervals for the PTT-BP relation when estimating PEP. The significant discrepancies are originated from the large PEP variability depicted in Figure 9B. The lower panel **(B)** highlights the SBP measurement uncertainty induced by estimating PEP with provided confidence intervals.

Although the proportion of PEP appeared to be constant, our data exposed a large PEP uncertainty when estimating the PEP from a relation between PEP and SBP. This was due to the large interindividual variability, both in PEP at rest and in PEP dynamic under increasing load. Applying the SBP specific standard deviation of PEP allowed us to create confidence intervals for a PTT-SBP relation when estimating PEP (Figure 10A). Accordingly, we were able to calculate the measurement uncertainty for measuring SBP when estimating PEP instead of directly measuring it. Notably, the specific PTT-SBP relation (Figure 9C) leads to increasing SBP measurement uncertainties with increasing SBP levels (Figure 10B).

Importantly, SBP measurement uncertainty increased even more when ignoring PEP instead of estimating it. The reasons for this were the large variability in resting PEP and interindividual discrepancies of PEP dynamic which are not at all accounted for when PEP is neither measured nor estimated.

## Discussion

In this present study, our results confirm the statement of low intraindividual PEP variability under resting conditions. Unfortunately, our findings do not support the claim that PEP plays a subordinate role in PWV-based applications (17). Quite to the contrary, we were able to show that PEP is a parameter of high interindividual variability which is prone to large changes under the influence of mental and physical load. These results confirm and extend the findings of psychobiologists, who demonstrated individual differences in PEP response to situations of perceived stress (20–22).

The ECG’s Q-wave is the physiologically coherent starting point of PEP as it symbolizes the beginning of ventricular depolarization. Historically, both the R-wave and the Q-wave have been used as starting markers for PWV applications (32, 33). Our results showed that the Q-R-time did not change with increasing heart rate and showed a low overall variability within subjects. This indicates that changes in PEP could be mainly due to changes in cardiac inotropy (shorter isovolumetric contraction) and less due to a dromotropic acceleration of signal transduction. As the effect of choosing the Q- or R-wave seemed to be negligible for determining changes in PEP, we decided on the ECG’s Q-wave as beginning of PEP.

We were able to show that PEP changes noticeably during mental (TSST) and physical (ergometer) stress. During the TSST, we observed the largest effect on PEP during the first measurement, likely representing the expected habituation effect (34). Under physical load, the PEP showed a clear load-response relationship. This could be explained by PEP being influenced by an increasing shift from parasympathetic to sympathetic activity of the autonomous nervous system. Accordingly, PEP showed a strong correlation with surrogate parameters of sympathetic tone, albeit with still noticeable discrepancies. This might indicate different regulatory patterns for PEP in contrast to heart rate, left-ventricular-ejection-time or SBP.

Important for any PWV-based application, PEP showed a pronounced interindividual variability at rest and even more distinct differences in response to mental or physical load. Consequently, PEP’s influence on PWV measurements is large and highly variable. Present PWV-based applications for BP measurement have shown promising measurement accuracy, even without directly measuring PEP (6, 10, 31, 33, 35). Notably, most well-performing devices rely on a BP calibration, which calibrates the device to the subject specific resting PEP (6, 8, 10). The calibration is effective for situation without big BP changes. Studies reporting large differences between reference and PWV-derived BP values might suffered from inadequate (e.g., not at total rest) calibration or could be due to severe problems with the cuff-based reference device (36, 37). However, such devices should also be prone to large measurement error in subjects with non-average PEP dynamic, who cannot be screened for beforehand. Supporting this, we were able to show the high heterogeneity in PEP behavior even in our very homogenous study population (Figure 8). Altogether, PEP might be one of the largest contributors to measurement uncertainty in cuff-less and continuous BP measurement.

Not only did we observe large PEP variability, but PEP’s correlation to heart rate changed dramatically under changing circumstances. While there was a very strong correlation under physical load, it weakened under mental stress and was almost non-existent under resting conditions. This is unfortunate, as the heart rate is the only easily and reliably detectable parameter of sympathetic balance which could have been a reasonable option for estimating PEP. Further, PEP was not the same under similar heart rates, when there was a change between rest, mental or physical load. Generally, PEP was lower under situations of mental or physical load compared to resting conditions.

While this makes it difficult to find a surrogate parameter for PEP, it enables exciting new possibilities for PEP-based applications. A model based on heart rate and PEP discriminated impressively well between rest, mental and physical load. There might be an opening for PEP-based stress monitoring with possible applications in psychology for treatment monitoring or mental self-care applications with focus on work-life balance or stress reduction. This is not to be neglected as both the demand for mental health services and rate of clinical diagnoses of mental health problems are rising steadily (38, 39).

Consequently, PWV-based applications are prone to very high measurement uncertainty when either neglecting or estimating PEP. We were able to investigate the latter visually, when subtracting a polynomial relationship between SBP and PEP derived from our data from a formerly published and robust PWV-SBP relation (6). Even though on average PEP seemed to be a constant proportion of PAT, the variability of PEP for all SBP level led to large confidence intervals of PTT. Therefore, the SBP measurement uncertainty induced by estimating PEP is very large, inflating to extreme values for high SBP due to the specific form of the published PWV-SBP relation. This effect can at least partly be mitigated by performing a calibration measurement and therefore account for the large interindividual differences in resting PEP. Albeit calibration cannot account for the differences in PEP dynamic, which were very prominent in our study and should only become more pronounced when applying any PWV-based BP measurement device to the much more diverse patient population in clinical practice.

All of this supports the claim that measuring PEP directly would be a valuable improvement for any ECG-based PWV application for measuring BP. Although we strongly support the broad integration of PEP into future models, it has to be acknowledged that there are well-performing PWV-based systems, not attributing for PEP (10, 33, 35). This may be due to PEP’s strong correlation to SBP, devices relying on calibration and cohort effects, which mitigate individual variability when assessing measurement performance over a large group of patients.

Our study is mainly limited by the homogenous study population of young and healthy subjects. This was done by choice as we aimed at first understanding and characterizing the PEP’s behavior in a physiological, disease-free scenario. Aging and disease might change the impact of PEP on PWV-based applications quite dramatically. In our population, PEP declined under increasing load, indicating that PEP-reducing factors (increase in cardiac inotropy) overwrite PEP-increasing factors like an increase in diastolic BP.

It is plausible to assume that this relation might change when the inotropic reserve declines with increasing age and morbidity (40, 41). At the same time, increased (vascular) age coincides with an increased PWV. Therefore, the absolute pulse-arrival-time measured in older patients is decreased (2, 42). Followingly, any change in PEP becomes a larger factor as its relative proportion compared to the pulse-transit-time increases. Consequently, the expected rise in PEP variability in a more diverse patient cohort further highlight the need of measuring PEP when trying to provide PWV-based applications which should be generalizable to a wide variety of patients, across all ages and medical conditions.

Prospectively, it is reasonable to assume that those existing systems will only improve when attributing for PEP properly. PWV-based applications for BP measurement assume that PWV is connected to arterial stiffness which itself correlates with SBP. Getting the electrical and isovolumetric periods of PEP out of an equation solely based on vessel stiffness makes sense, conceptionally, physiologically, and mathematically.

Lastly, enabling the continuous detection of PEP for wearable devices would open the door for more advanced systems. Impedance cardiography provides multiple parameters, many of which are most likely predictive for BP (e.g., left-ventricular-ejection-time, Heather index, acceleration index, etc.). Creating a model based on multiple predictive parameters, ideally including PWV and PEP, could be an important step in the direction of reliable and convenient non-invasive, cuff-less, and continuous BP measurement.

## Conclusion

The PEP is a stress-dependent parameter of high importance for PWV-based applications for BP measurement. While it shows little intraindividual variability under resting conditions, it is highly modulated by mental and physical load. We were able to show that the interindividual variability is profound, when analyzing both resting values and stress modulated PEP dynamic.

Consequently, PWV-based applications cannot ignore PEP without accepting considerable measurement uncertainties. Our study revealed that PEP has a strong correlation to parameters of sympathovagal balance, but also that the strength of correlation is highly stress-modulated and barely existing under resting conditions. Therefore, estimating PEP *via* a surrogate such as the heart rate is not feasible. The effects of neglecting or even estimating PEP for PWV-based SBP measurement applications is considerable. Our experiment revealed serious measurement uncertainties caused by estimating PEP instead of measuring it.

Concludingly, measuring PEP directly offers the chance of greatly improving PWV-based systems for BP measurement, even under already well-performing circumstances. Further, continuous impedance cardiography might enable more complex and capable systems in the future.

## Data availability statement

The raw data supporting the conclusions of this article will be made available by the authors, without undue reservation.

## Ethics statement

The studies involving human participants were reviewed and approved by the Local Ethics Committee of Charité–Universitätsmedizin Berlin, approval-number: EA4/051/21. We registered the study at Charité–Universitätsmedizin Berlin’s clinical trial register before the start of data collection (ePA: 3000224). The patients/participants provided their written informed consent to participate in this study.

## Author contributions

NP carried out the majority of the experimental work and wrote the manuscript. TB and NP performed the statistical analysis. AP provided valuable advice on the design and conduct of the study. All authors contributed to the article and approved the submitted version.
