# Supplementary material for: The pre-ejection period is a highly stress dependent parameter of paramount importance for pulse-wave-velocity based applications
Source: Front Cardiovasc Med. 2023 Feb 15;10:1138356. doi: 10.3389/fcvm.2023.1138356 (PMC9975268; doi:10.3389/fcvm.2023.1138356)
Supplement: Supplementary file 1 [file Data_Sheet_1.docx]

**Supplement 1: Adapted Trier Social Stress Test**


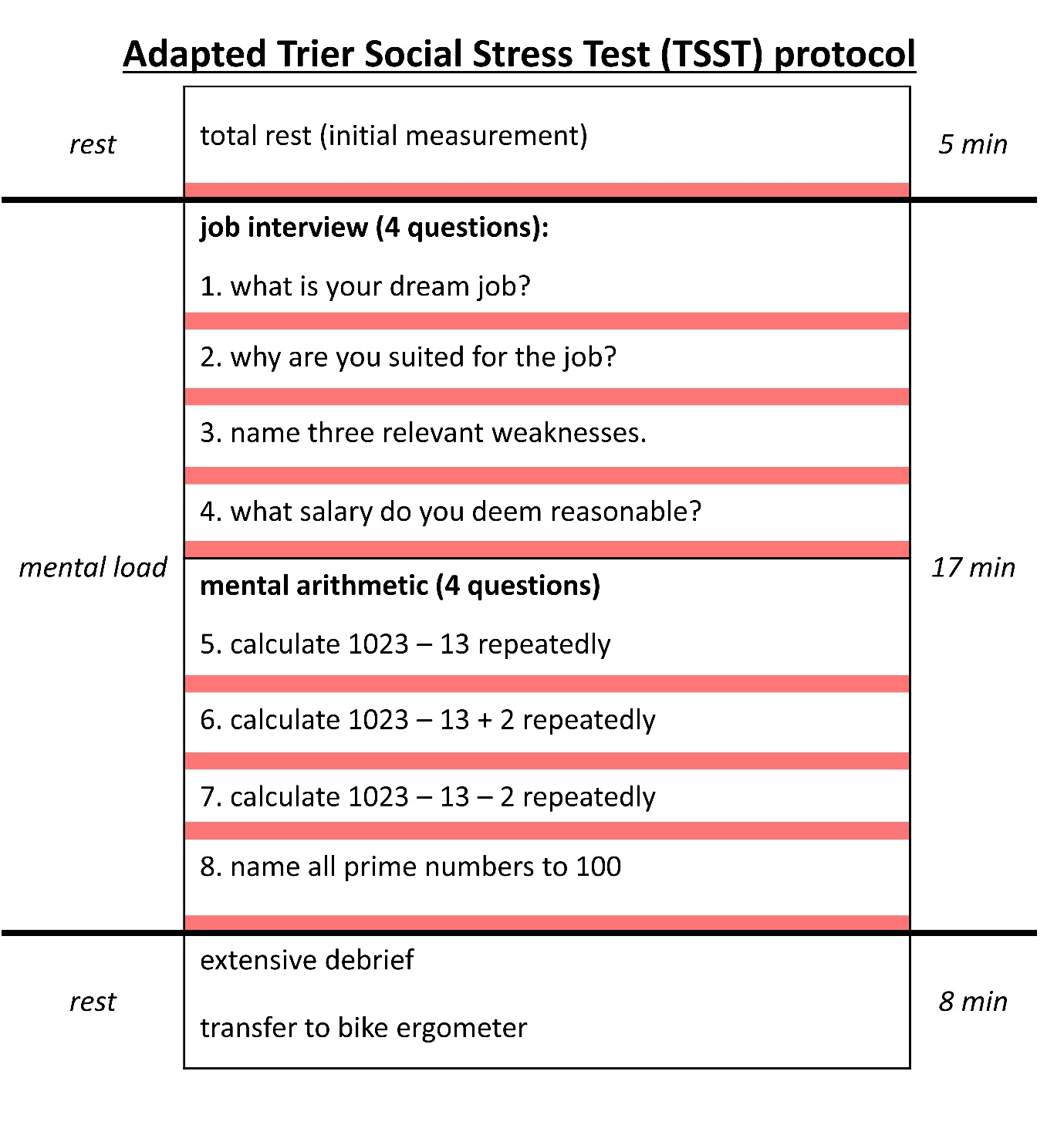

*Supplement 1: Adapted Trier Social Stress Test (TSST) protocol: Experimental protocol of the adapted TSST performed in the experiment. Blood pressure measurements are indicated by the red lines.*

After the measurement at total rest was performed, a researcher dressed in a white lab-coat sat down at a table 3 meters away but in direct eyesight of the participants. The researcher introduced himself as the “investigator” who will lead the experiment from now on. Followingly, the investigator reveals to the participants that they would simulate a job application interview. He then positioned a clearly visible clock and a fake (unknown to the participants) camera in front of the participants. The participants were then instructed that the investigator will read job interview questions to them which they should answer within 60 seconds while only speaking to the camera.

It was stressed to them that they must not stop talking during the 60 seconds but that they need to stop talking immediately after the instructor indicated them that the time ran out. After each answered question, we took a BP measurement during which participants were instructed to remain silent. The investigator read the next question to the participants while we performed the BP measurement. The participants were informed that they must start talking immediately after the instructor finished asking the question and the BP measurement had finished. Four questions were asked in imperative form:

1. Explain the job of your dreams in 60 seconds.
2. Explain why you are better suited for the described position than people in your personal surrounding.
3. Outline three weaknesses of yours, relevant to the desired position.
4. Explain which salary you seem reasonable for the desired position. Keep in mind that the HR manager, who will evaluate this interview, has a salary of about 3,200€ before taxes.

After participants answered the fourth question, the investigator indicated that they would continue with mental arithmetic questions in the same fashion. Again, the participants had 60 seconds each to give answers to the tasks. The investigator informed them, that they would have to start again from the beginning if an error occurred. The investigator did not elaborate what kind of error was made and instructed the participants to start again without further information if participants failed to give the correct answer. The investigator gave four tasks to the participants:

1. Subtract 13 from 1023 repeatedly: [1023, 1010, 997, …]
2. Subtract 13 and then add 2 to 1023 repeatedly: [1023, 1010, 1012, 999, …]
3. Subtract 13 and then 2 from 1023 repeatedly: [1023, 1010, 1008, 995, …]
4. Name all prime numbers smaller than 100 in ascending fashion: [2, 3, 5, 7, …]

The investigator avoided eye contact during the full duration of the adapted TSST to ensure standardized circumstances and deprive participants from social support.

After participants answered the last question, we performed a final BP measurement. All participants received an extensive debriefing after the first experimental phase in which we revealed that there was no recording and that the experiment was solely designed to exert mental stress on them. For the debrief, the investigator undressed the lab coat and engaged in and open and friendly social situation.
